# Comparison of the Chemical Composition of Different Body-Color Phenotypes of *Perinereis aibuhitensis* (Grube, 1878) (Annelida, Nereididae)

**DOI:** 10.3390/biology15090706

**Published:** 2026-04-30

**Authors:** Huan Liu, Jiahao Liu, Chenchen Bian, Qiang Ma, Yuliang Wei, Mengqing Liang, Houguo Xu

**Affiliations:** 1 State Key Laboratory of Mariculture Biobreeding and Sustainable Goods, Yellow Sea Fisheries Research Institute, Chinese Academy of Fishery Sciences, Qingdao 266071, China; 2 Laboratory for Marine Fisheries Science and Food Production Processes, Qingdao Marine Science and Technology Center, Qingdao 266237, China

**Keywords:** clamworm, pigmentation, reproduction, aquaculture, feed, functional food

## Abstract

A comparative chemical analysis was conducted between *P. aibuhitensis* of orange and green body colors, evaluating their proximate composition, fatty acid profile, amino acid profile, astaxanthin content, lipidomic profile, and other biochemical parameters. Samples were categorized by body color into two groups, each with ten biological replicates. The samples were collected from the same local polychaete farm. The results revealed that the green phenotype had significantly higher moisture content but lower crude protein, crude lipid, and ash content compared to the orange phenotype. The orange polychaete was characterized by significantly higher concentrations of 16:0 and saturated fatty acids (SFAs), whereas the green one exhibited higher contents of n-6 polyunsaturated fatty acids (n-6 PUFAs) and a higher PUFA/SFA ratio. Regarding free amino acids, the orange polychaete had significantly higher threonine content, while the green ones had significantly higher levels of valine, isoleucine, leucine, phenylalanine, glutamate, alanine, histidine and proline. Additionally, the astaxanthin content was significantly higher in the orange phenotype. The bile acid level was significantly higher in the green phenotype compared to the orange one, but no significant differences were observed in other biochemical parameters such as total protein, total cholesterol, and triglyceride content. The lipidomic analysis revealed that glycerophospholipids were the most abundant lipid class in both phenotypes, followed by glycerolipids and sphingolipids. A total of 65 differentially abundant lipid molecules were identified between the two groups. Compared to the orange polychaete, the green one had higher levels of 59 lipids (predominantly ceramides) and lower levels of six lipids, including three triglycerides, one monogalactosyldiacylglycerol, and two phosphatidylserines. In general, the orange *P. aibuhitensis* showed a favorable nutritional profile for aquafeed and human nutrition, whereas the green ones had potential for targeted health applications owing to its specific lipid composition. However, direct validating experiments are required.

## Simple Summary

Stable color phenotypes are commonly observed in polychaete *Perinereis aibuhitensis*. However, the nutritional implications of this phenotypic variation are still poorly understood. This study compared the nutritional components of polychaete with two body colors, orange and green. The results showed that the orange polychaete had higher contents of crude protein, crude lipid, astaxanthin, and saturated fatty acids, as well as a more favorable ratio of n-3/n-6 polyunsaturated fatty acids, suggesting a favorable nutritional profile that may be suitable for human nutritional supplements and aquaculture feeds. The green polychaete was rich in ceramides, bile acids, and some umami amino acids, showing potential for the development of specific functional products. This study suggests that body color can serve as an important indicator of nutritional quality in polychaetes.

## 1. Introduction

The polychaete (*Perinereis aibuhitensis*) (Grube, 1878) is a marine benthic organism belonging to the phylum Annelida, class Polychaeta, order Errantia, and family Nereidae. It is widely distributed in the Bohai Sea, East China Sea, and South China Sea [1]. *P. aibuhitensis* is highly nutritious, characterized by its high protein content and abundance of essential amino acids, PUFAs, and bioactive compounds (such as astaxanthin) [2,3]. These nutritional qualities make it valuable feed in aquaculture and even good food for human consumers. The application of *P. aibuhitensis* in aquafeeds, alone or as a supplement in formulated feeds, has been demonstrated to promote the gonadal development and spawning performance of broodstocks [4], increase palatability [5], and replace fishmeal without compromising fish growth [6,7]. For human consumers, the polychaetes are also potential functional foods or nutritional supplements, providing essential nutrients, antioxidant properties, and metabolic regulation benefits. In some provinces of China, the polychaete has long been a traditional high-grade food in the history. For example, *Tylorrhynchus heterochaetus*, widely distributed in the coastal regions of Guangdong, Guangxi, Zhejiang, and Fujian provinces in China, has a long history as human food and exhibits antioxidant, anti-inflammatory, and anti-fatigue effects, making it a promising candidate for functional foods and pharmaceuticals [8]. In addition, *Neanthes japonica* is also considered to have nutritional value for human consumption due to its high-quality nutrients [9].

Commonly, *P. aibuhitensis* has an orange body color. However, varieties with green body color also widely exist. During the early developmental stage, no different body color is observed. However, when individuals reach approximately 0.5 to 1.0 cm in length, *P. aibuhitensis* begins to exhibit distinct color variations, forming individuals with clearly different body colors that can be stably inherited [10].

Existing comparative studies on the chemical or nutritional composition of polychaete have mainly focused on the comparison between geographical variations [11,12] or that between wild and farmed populations [13]. However, systematic research into the nutritional differences between color phenotypes remains limited. Therefore, this study was aimed at comprehensively comparing the nutritional compositions of orange and green *P. aibuhitensis*, in terms of proximate composition, fatty acid and amino acid composition, biochemical indicators, astaxanthin content, and lipidomics. The findings are expected to be helpful in deeply understanding the nutritional characteristics of *P. aibuhitensis*, and consequently be able to support the comprehensive evaluation of its potential as functional food or nutritional supplement for both aquatic animals and human consumers.

## 2. Materials and Methods

### 2.1. Experimental Materials

**Figure 1:**
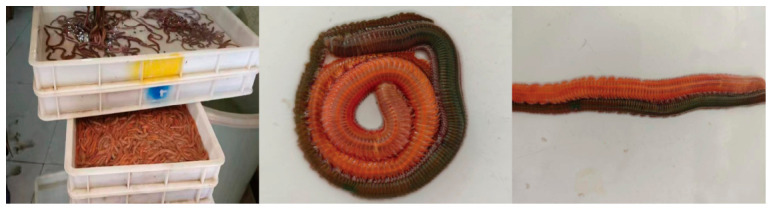
The two body colors (orange and green) of *P. aibuhitensis*.

The *P. aibuhitensis* samples were collected from a local aquaculture farm in Jimo (Qingdao, Shandong Province, China). The *P. aibuhitensis* specimens were cultured in outdoor seawater ponds. Healthy specimens with intact bodies and high vitality were selected, with body lengths ranging from 15 to 25 cm (Figure 1). All samples used in this experiment were adult *P. aibuhitensis* reared under identical culture conditions.

### 2.2. Sample Collection

Based on body color, the samples were divided into two groups, each comprising ten biological replicates. All samples were washed in clean water 2~3 times. For each replicate, six polychaetes with consistent body color were randomly selected and pooled to form a single sample for subsequent chemical analysis. Due to the high cost of lipidomics analysis, a pooling strategy was adopted to control experimental costs. Specifically, ten biological samples were equally pooled and then aliquoted into three parallel samples for subsequent analysis. The research protocol, including the research question, key experimental design, and data analysis plan, was finalized prior to the start of the study. The authors confirm that the ethical policies of the journal have been adhered to. This study did not require ethical approval as polychaetes are not subject to animal welfare regulations.

### 2.3. Analytical Methods

#### 2.3.1. Proximate Composition

The proximate composition was analyzed according to AOAC (2005) standards [14]. For the analysis of moisture, samples were dried in a 105 °C oven until constant weight. Dried samples were ground and used for the analysis of crude protein, crude lipid, and ash content. The crude protein content was quantified using the Kjeldahl method (FOSS Soxtec 2050, FOSS A/S, Hillerød, Denmark), the crude lipid content by the Soxhlet extraction method (WY-SXT-06, Shandong Wuyue Instrument Co., Ltd., Jinan, China), and the ash content by the high-temperature (550 °C for 8 h) combustion method in a muffle furnace (SX2-4-10, Longkou Electric Furnace Manufacturing Factory, Yantai, China).

#### 2.3.2. Fatty Acids

Total lipids were extracted using a chloroform–methanol protocol, and then the fatty acid composition was analyzed by gas chromatography. Briefly, 2 mL KOH–methanol was added to the sample and the solution was incubated at 75 °C for 30 min. After cooling, 1 mL boron trifluoride–methanol solution was added and mixed by vortex. The mixture was again incubated at 75 °C for 30 min, cooled, and then supplemented with 1 mL hexane and 1 mL purified water, followed by incubation on ice for 1 h. The supernatant was collected after centrifugation at 1308× *g* for 2 min, filtered through a membrane, and injected into a gas chromatograph (GC-2010 Pro, Shimadzu Corporation, Kyoto, Japan). Results are expressed as relative percentages of total fatty acids.

#### 2.3.3. Amino Acids

Amino acid profiles were assayed using an automatic amino acid analyzer (L-8900, Hitachi, Ltd., Tokyo, Japan). For total amino acids, 0.02 g freeze-dried sample was hydrolyzed with 15 mL HCl (6 mol/L) at 110 °C for 24 h. The hydrolysate was diluted to 50 mL, and 0.5 mL was selected and dried under nitrogen at 40 °C. The residue was reconstituted in 1 mL HCl (0.02 mol/L), filtered, and analyzed. For free amino acids, 0.02 g freeze-dried sample was treated with 1.6 mL trichloroacetic acid (6%), vortexed, sonicated, and centrifuged. The supernatant was deacidified at 90 °C, redissolved in HCl (0.02 mol/L), filtered, and analyzed. Separation was achieved via ion-exchange chromatography with elution using pH-graded buffers, followed by post-column derivatization with ninhydrin for detection at 570 nm.

#### 2.3.4. Astaxanthin

Astaxanthin content was analyzed by Qingdao Yuanxin Technology Testing Technology Co., Ltd. (Qingdao, China). The analysis method was performed according to the Chinese industry standard SC/T 3053-2019 [15].

#### 2.3.5. Other Biochemical Parameters

The concentrations of crude protein, triglycerides, cholesterol, and bile acids were assayed using commercial assay kits (Nanjing Jiancheng Bioengineering Institute, Nanjing, China) according to the manufacturer’s instructions.

#### 2.3.6. Lipidomics

Lipids were extracted from 50 mg sample using a methanol/water and methyl tert-butyl ether (MTBE) mixture. The homogenate was sonicated at 5 °C, incubated at −20 °C, and then centrifuged. The upper lipid layer was collected, dried under nitrogen, and reconstituted in isopropanol/acetonitrile (1:1, *v*/*v*). The lipidomic analysis was performed using a Thermo UHPLC-Q Exactive HF-X system (Thermo Fisher Scientific, Waltham, MA, USA) equipped with an Accucore C30 column (100 mm × 2.1 mm, 2.6 μm). Mobile phases consisted of (A) 10 mM ammonium acetate in acetonitrile/water (1:1, *v*/*v*) with 0.1% formic acid, and (B) 2 mM ammonium acetate in acetonitrile/isopropanol/water (10:88:2, *v*/*v*/*v*) with 0.02% formic acid. The injection volume, flow rate, and column temperature were 2 μL, 0.4 mL/min, and 40 °C, respectively. Mass spectrometry was conducted using electrospray ionization in both positive and negative modes with data-dependent acquisition over *m*/*z* 20–2000. Equal volumes of all sample metabolites were mixed to prepare the quality control (QC) samples. During the instrument analysis process, one QC sample was inserted every ten samples. Raw data were processed using LipidSearch software (version 4.2, Thermo Fisher Scientific, Carlsbad, CA, USA) and the Majorbio Cloud Platform (cloud.majorbio.com). Lipidomics profiling was performed by Majorbio Bio-Pharm Technology Co., Ltd. (Shanghai, China).

### 2.4. Data Analysis

The experimental data were analyzed by independent sample *t*-tests with SPSS 25.0 statistical analysis software. *p* value < 0.05 was considered statistically significant, and data values are expressed as mean ± standard error.

For the lipidomics data, variables with relative standard deviation (RSD) > 30% of QC samples were removed. The principal component analysis (PCA) was performed with R package (Version 1.6.2). A 7-cycle interactive validation was done to evaluate the stability of the model. Lipids with a fold change (FC) ≥ 1.2 and a *p* < 0.05 from *t*-test were considered significantly different between the two groups.

## 3. Results

### 3.1. Proximate Composition

Significant differences in the body composition were observed between the two color phenotypes (Table 1). Compared to the orange polychaete, the green phenotype had significantly higher moisture content, but significantly lower levels of crude protein, crude lipid, and ash (*p* < 0.05).

### 3.2. Fatty Acid Compositions

**Figure 2:**
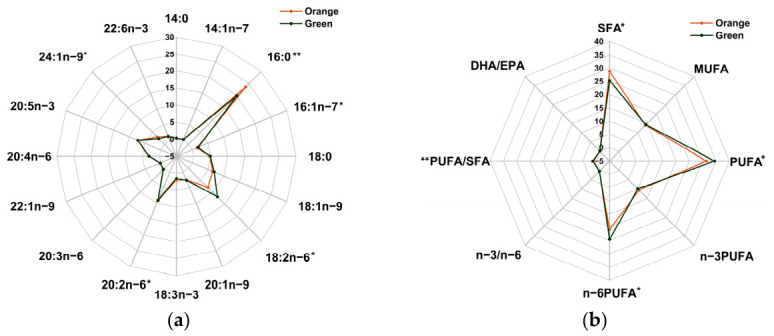
(**a**) Individual fatty acid composition (% total fatty acids) of the orange and green *P. aibuhitensis*. (**b**) Summary of fatty acid class profiles (% total fatty acids) and key functional ratios of the orange and green *P. aibuhitensis*. * and ** mean significant differences between the orange and green *P. aibuhitensis*, and the significance levels are *p* < 0.05 and *p* < 0.01, respectively.

Compared to the orange *P. aibuhitensis*, the green phenotype showed significantly lower levels of SFAs (*p* < 0.05), with higher PUFA, n-6PUFA, and PUFA/SFA (*p* < 0.05; Figure 2a) levels. Compared to orange *P. aibuhitensis*, green ones had lower levels of ARA, EPA, and DHA, but the differences were not statistically significant (*p* > 0.05). The contents of 16:0 and 24:1n-9 in green *P. aibuhitensis* were significantly (*p* < 0.05) lower, while those of 16:1n-7, 18:2n-6 and 20:3n-6 were significantly (*p* < 0.05) higher compared to orange ones (Figure 2b).

### 3.3. Amino Acid Compositions

**Figure 3:**
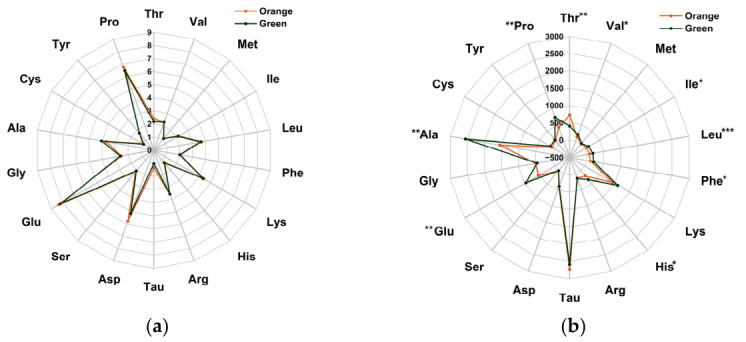
(**a**) Total amino acid composition of the orange and green *P. aibuhitensis* (% dry matter basis); (**b**) free amino acid composition of the orange and green *P. aibuhitensis* (mg/100 g dry matter). *, **, *** mean significant differences between the orange and green *P. aibuhitensis*, and the significance levels are *p* < 0.05, *p* < 0.01, and *p* < 0.001, respectively.

No significant differences were observed in total amino acid content between the two phenotypes (*p* > 0.05; Figure 3a). However, for free amino acids, the green phenotype had significantly lower Thr content (*p* < 0.05), but higher contents of Val, Ile, Leu, Phe, His, Glu, Ala, and Pro than the orange phenotype (*p* < 0.05; Figure 3b).

### 3.4. Astaxanthin Content

**Figure 4:**
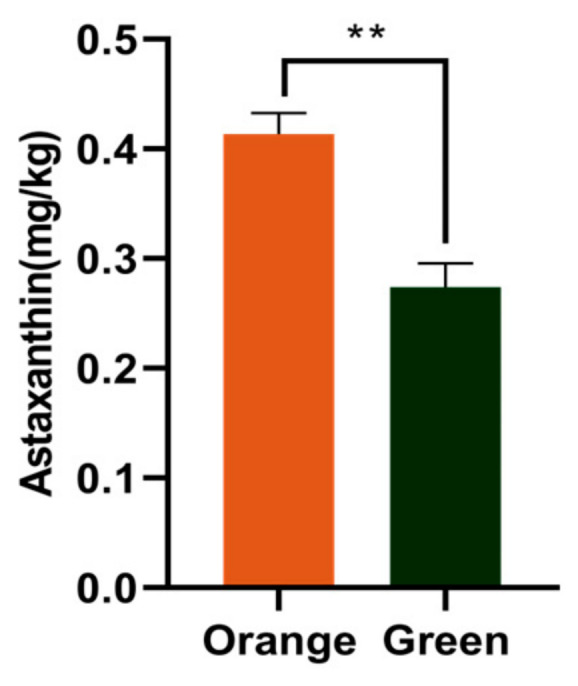
The content of astaxanthin of the orange and green *P. aibuhitensis* (mg/kg wet weight). ** means significant difference between the orange and green *P. aibuhitensis*, and the significance level is *p* < 0.01.

Compared to orange *P. aibuhitensis*, the green ones had significantly lower astaxanthin content (*p* < 0.05; Figure 4).

### 3.5. Other Biochemical Compositions

**Figure 5:**
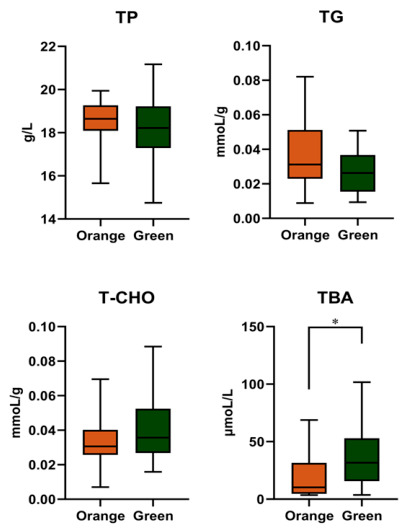
The biochemical indicators of the orange and green *P. aibuhitensis*. * means significant difference between the orange and green *P. aibuhitensis*, and the significance level is *p* < 0.05.

There were no significant differences in total protein, total cholesterol, and triglyceride content between the two color phenotypes (*p* > 0.05). However, the bile acid content was significantly higher in the green phenotype compared to orange (*p* < 0.05; Figure 5).

### 3.6. Lipidomics

#### 3.6.1. General Description

The lipidomic analysis identified a wide range of lipid molecules, which were across five major categories: GP, GL, SP, FA, and ST. GP had the highest content, accounting for 50.13% of total lipids, followed by GL and SP, which accounted for 25.50% and 18.59%, respectively (Supplementary Figure S1). A total of 48 lipid subclasses were identified, containing 1506 lipid molecules. The 48 subclasses include 20 GP, 5 GL, 12 SP, 6 FA, and 5 ST. Specifically, TG (16.93%), PE (16.73%), PC (13.21%), and Cer (12.48%) are the most abundant ones. TG and PE contain the most diverse types of lipid molecules, with 255 and 252 types, respectively (Supplementary Table S1).

#### 3.6.2. Multivariate Statistical Analysis of Lipid Molecules

**Figure 6:**
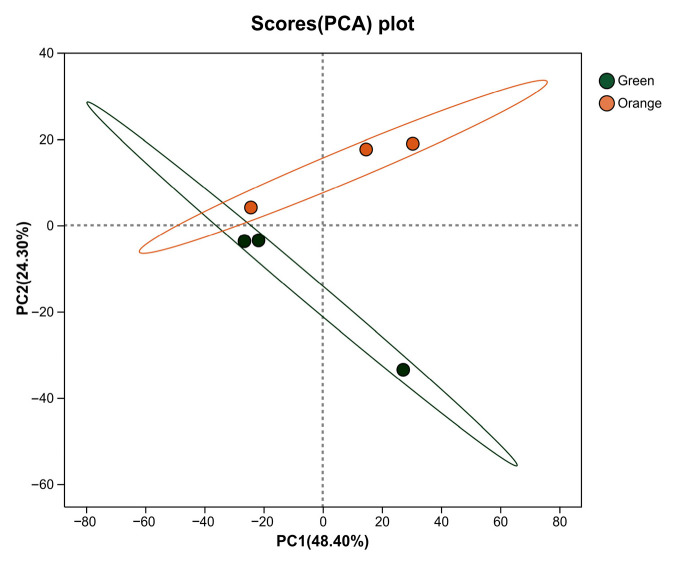
The principal component analysis (PCA) model of the orange and green *P. aibuhitensis*. The green and orange points correspond to green and orange *P. aibuhitensis* individuals, respectively.

Principal component analysis (PCA) was employed to visualize group separation and identify differentially abundant lipids, and the result showed that the two phenotypes were generally separated in the PCA plot (Figure 6).

#### 3.6.3. Significantly Different Lipid Molecules in Abundance Between Different Body-Color Phenotypes

**Figure 7:**
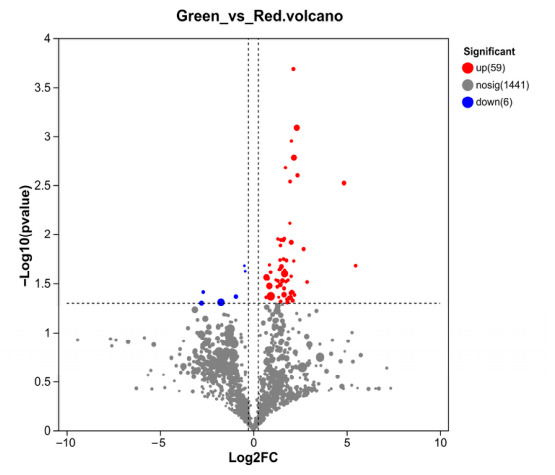
Volcano plot of differentially abundant lipid molecules between orange and green *P. aibuhitensis*. The red and blue dots represent significantly upregulated and downregulated lipids in green *P. aibuhitensis* compared to orange individuals, respectively. Significantly different lipids were defined as fold change (FC) ≥ 1.2 and *p* < 0.05 (*t*-test). The horizontal dashed line marks the significance threshold of *p* = 0.05, and the vertical dashed lines mark the fold change threshold of FC = 1.2. Gray dots represent non-significant lipids.

**Figure 8:**
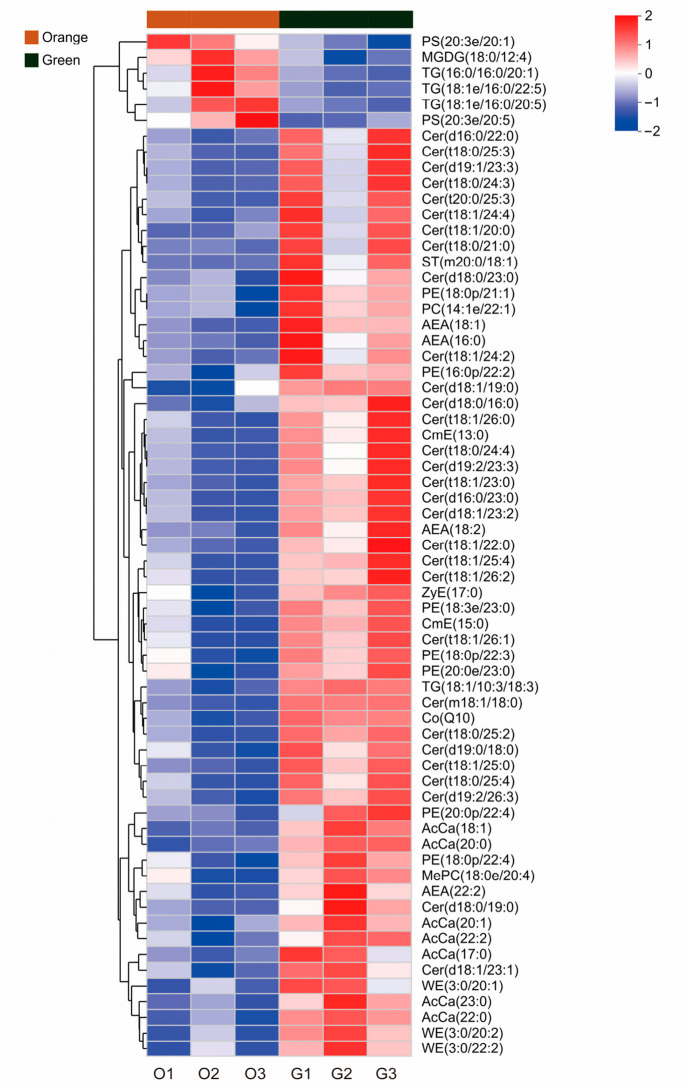
Heatmap of differentially abundant lipid molecules of the orange and green *P. aibuhitensis*. The average concentration of a certain lipid metabolite was standardized to be 0. Higher concentration than the average was labeled as red, and lower concentration was labeled as blue. The color value as indicated in the right color bar means fold of standard deviation distant to the average concentration. O: the orange *P. aibuhitensis*. G: the green *P. aibuhitensis*.

A total of 65 lipid molecules were different in abundance between the two groups. Compared to the orange phenotype, six lipid molecules in the green phenotype had lower abundance, which consisted of three TG, two PS and one MGDG, and 59 had higher abundance, with the majority (30 out of 59) belonging to the Cer class; other lipid molecules included seven PE, seven AcCa, four AEA, three WE, two CmE, and one ST, PC, ZyE, TG, CO and MePC. (Figure 7 and Figure 8).

## 4. Discussion

*P. aibuhitensis* has long been used as human food and in fish broodstock diets, but most reports have not specified the body color of the samples used [3,16]. In fact, variations in body color of aquatic animals, which widely exist in nature, are often closely related to differences in nutritional components [17,18]. Specific to *P. aibuhitensis*, the body color variation commonly happens in both wild and farmed populations. However, the chemical composition of varieties with different body colors has not been comprehensively evaluated. This is the first study comparing the chemical composition of *P. aibuhitensis* with different body colors.

It is also important to clarify that according to previous studies, the body color variation in *P. aibuhitensis* is not sex-dependent; both orange and green body colors were found in both females and males [10]. Therefore, sex distribution was likely balanced between the two color groups and did not systematically bias the comparisons. Moreover, during sexual maturation, *P. aibuhitensis* undergoes metamorphosis, transforming from normal morphology into reproductive bodies (heteronereis). During this period, changes happen such as shortening of body trunk, whitening of abdomen, enlargement of eyes, and thinning of parapodia. The heteronereids die after reproduction [19]. All specimens used in this study were adults with normal external morphology, without any signs of these maturation-related features. While minor effects of developmental status on biochemical composition cannot be completely excluded, the morphological evidence and existing literature support body color as the primary factor driving the observed different phenotypes.

### 4.1. Proximate Composition

The proximate composition of polychaetes was the foremost concern for both human consumption and aquafeed. In this study, compared to the orange *P. aibuhitensis*, the green ones had higher moisture content, but lower contents of crude protein, crude lipid, and ash, implying reduced nutritional value [20]. It has been revealed that the proximate composition of polychaete varies significantly across different regions [12,16]. Therefore, both region and body color can serve as important indicators of nutritional quality of *P. aibuhitensis*, which needs to carefully considered in practical application.

### 4.2. Fatty Acid Composition

Beyond proximate composition, *P. aibuhitensis* contains abundant PUFAs, especially EPA, DHA, and ARA [3]. In human, these fatty acids are beneficial for cardiovascular health [21,22], brain development [23], and anti-inflammatory effects [24]. For aquatic animals, these fatty acids play additional important roles not only in gonadal development, fecundity, spawning rate, and hatching rate of broodstocks [25,26], but also in growth performance and health status of both grow-out and juvenile fish [26,27,28]. More importantly, polychaetes are among the few aquatic animals capable of synthesizing LC-PUFAs on their own, providing an additional avenue for obtaining these valuable LC-PUFA resources [29,30,31,32,33,34]. It is noteworthy that while the LC-PUFA biosynthetic capability has been well documented in several nereidid polychaetes, such as *Namalycastis rhodochorde* [29], *Hediste diversicolor* [31,34], *Platynereis dumerilii* [33], etc., direct experimental evidence for such capacity in *P. aibuhitensis* is currently lacking. Given its phylogenetic proximity within the family Nereididae, it is highly plausible that *P. aibuhitensis* also possesses a similar enzymatic machinery for LC-PUFA synthesis. However, this study found no significant differences in EPA, DHA and ARA between the orange and green phenotypes. For saturated fatty acids, the orange *P. aibuhitensis* had significantly higher levels of 16:0 than the green phenotype. For fish and shrimp broodstocks, as a preferential substrate for β-oxidation, 16:0 accumulates during ovarian development and provides energy for embryogenesis [35]. A study on American shad (*Alosa sapidissima*) revealed that the content of 16:0 in the ovaries increased significantly as the female broodstock’s ovaries developed [35]. On the other hand, the higher SFA content in the orange *P. aibuhitensis* has been associated with increased risks of cardiovascular disease [36], insulin resistance, and type 2 diabetes [37] in humans. Regarding MUFA, the green *P. aibuhitensis* contained significantly more palmitoleic acid (16:1n-7), which has potential anti-inflammatory, antidiabetic, and cardioprotective effects [38], as well as skin-whitening effects [39].

Besides individual fatty acids, the n-3/n-6 PUFA ratio is a key regulatory factor in aquatic animal nutrition, influencing the entire life cycle and exerting systemic effects on reproduction, development, growth, health, and physiological regulation. An appropriate n-3/n-6 ratio promotes gonadal development and offspring quality, whereas an imbalanced ratio reduces reproductive success [40]. For example, in Nile tilapia (*Oreochromis niloticus*), a lower n-6/n-3 PUFA ratio is associated with higher ovarian aromatase gene expression and better spawning performance [41]. Specifically, live feeds with an n-3/n-6 ratio closer to that in mature shrimp ovaries lead to better spawning outcomes of shrimp [42]. In the mature ovaries of *L. vannamei*, the n-3/n-6 PUFA ratio is 0.67 [43]. The present study showed that the n-3/n-6 PUFA ratio in the orange *P. aibuhitensis* (0.51) is closer to this value compared to the green phenotype (0.41), suggesting a potential advantage of the orange phenotype for fish or shrimp broodstock reproduction. Previous feeding experiments have indirectly supported that broodstock fed orange *P. aibuhitensis* showed higher mating rates, gonadosomatic index (GSI), and hepatosomatic index (HSI) [44]. Nevertheless, direct comparative feeding trials using different color phenotypes are necessary to confirm this hypothesis. While the n-3/n-6 ratio plays a crucial role in broodstock reproduction, its significance for the growth performance and immune health of aquatic animals should not be overlooked. An optimal n-3/n-6 PUFA ratio is equally vital for growth, as it enhances growth performance by regulating lipid metabolism, hepatic antioxidant capacity, and intestinal health [45]. Supporting this, Gomes [46] reported that in larvae of the Mediterranean purple sea urchin (*Paracentrotus lividus*), dietary supplementation with microalgae possessing a higher n-3/n-6 ratio resulted in significantly promoted larval growth compared to those fed with microalgae of lower ratios.

### 4.3. Astaxanthin Content

In addition to more balanced fatty acid ratio, the orange *P. aibuhitensis* is richer in astaxanthin than green ones, which probably contributes to their body color differences. For aquafeed, astaxanthin serves as not only a crucial pigmentation substance, but also strong antioxidant [47]. Polychaetes cannot synthesize astaxanthin endogenously. The astaxanthin present in their bodies is derived from dietary intake. This externally sourced astaxanthin undergoes esterification with the abundant endogenous LC-PUFAs in *P. aibuhitensis*, forming astaxanthin monoesters or diesters. This esterification process enhances the stability of astaxanthin within the organism [48]. Similarly, the astaxanthin content in Atlantic salmon (*Salmo salar*) muscle was positively correlated with EPA and DHA concentrations and negatively correlated with n-6 fatty acid concentrations [49]. In noble scallops (*Chlamys nobilis*) with different shell colors, total carotenoid content is significantly positively correlated with PUFAs and significantly negatively correlated with SFAs [50]. In this study, the orange *P. aibuhitensis* exhibited higher levels of SFAs and lower levels of n-6 PUFAs, which may facilitate the deposition or pigmentation efficiency of astaxanthin, although the precise mechanism requires further investigation.

Besides the pigmentation function, the antioxidative activity of astaxanthin is beneficial to both human consumers and the aquaculture industry, in particular broodstock management and larval rearing [51,52]. According to the European Food Safety Authority (EFSA), a daily intake of 8 mg of astaxanthin from supplements is considered safe for adults [53]. Assessing the actual astaxanthin content and bioavailability in edible parts of the orange *P. aibuhitensis* is essential for developing functional foods or additives. Dietary astaxanthin increases the yolk protein content of broodstock shrimp, fertilized egg hatching rate, larval metamorphosis rate, and the number of nauplii and zoea [54]. Similar results have been reported in the long snout seahorse (*Hippocampus guttulatus*) [55], rainbow trout (*Oncorhynchus mykiss*), [56] and Atlantic cod (*Gadus morhua*) [57]. In terms of antioxidation, astaxanthin is widely used as a potent antioxidant to mitigate oxidative stress, enhance immune function, and improve overall health status [58,59]. Similar results were also found in larval and post-larval kuruma shrimp (*Marsupenaeus japonicus*) [52]. The higher astaxanthin content in the orange *P. aibuhitensis* suggests a potential for health promotion, given the well-established antioxidant properties of astaxanthin. However, the actual bioavailability and functional effects in humans or aquatic animals require direct investigation.

### 4.4. Amino Acid Composition

Regarding amino acid profile, no significant difference was observed in the total amino acid content between the orange and green *P. aibuhitensis*. However, differences were observed in their content of free amino acids, which are increasingly recognized to make an important contribution to palatability and are important precursors for the formation of aroma compounds [60]. In terms of flavor, free amino acids can be categorized into umami amino acids, sweet amino acids, and bitter amino acids [61]. The orange *P. aibuhitensis* had significantly higher levels of the sweet amino acid (threonine), while the green one had more umami (Glu), sweet (Ala), bitter (Ile, Leu, Phe, His) and sweet/bitter (Val, Pro) amino acids. Although the green *P. aibuhitensis* had higher umami and sweet amino acid content, its higher bitter amino acid content may partially offset this advantage or even produce a negative impact, potentially affecting palatability for aquatic animals and human flavor acceptance. The observed differences in flavor-related amino acids may provide a clue for future studies on feeding preference.

### 4.5. Lipidomics

The lipidome analysis revealed that TG, PE, PC, and Cer are the major lipid classes in *P. aibuhitensis*. TG, PE and PC are the primary lipids in marine organisms [62,63], with TG serving as a key energy source [64] and phospholipids as the predominant lipids in shrimp ovaries (PC, 75–80% and PE, 20–25%) [42]. Compared to the green *P. aibuhitensis*, three out of the six significantly upregulated lipid molecules in the orange phenotype belong to TG. Although elevated triglyceride levels are generally associated with excessive fat deposition in aquatic animals [65], they serve as important functional substances in broodstock development research. High TG levels accumulate in the ovaries of mud crab (*Scylla paramamosain*), increasing significantly in the hepatopancreas and ovaries during maturation [66]. TGs are the primary energy reserve in shrimp fertilized eggs, transferred from the ovaries for egg hatching and nauplius metamorphosis [67]. Other research has shown that there was a transfer of triglycerides between the Pacific white shrimp (*Litopenaeus vannamei*) broodstock and offspring generations [68].

In contrast to TG, the green *P. aibuhitensis* had higher abundance of ceramide. Ceramides are a class of complex sphingolipids located at the center of sphingolipid biosynthesis and metabolism [69], which are the main lipids that constitute the stratum corneum [70]. For humans, the most widespread application of ceramides currently remains largely confined to the field of topical skincare products [71] with their potential role in nutritional health yet to be thoroughly investigated and reported. For aquatic animals, ceramides are also involved in the regulation of feed intake in rainbow trout [72], and can serve as key lipid markers indicating the clams (*Meretrix petechialis*) infected with *Vibrio* species [73] and the Mozambique tilapia (*Oreochromis mossambicus*) infected with pansteatitis [74]. The content of ceramides in fish can be regulated by other nutrients in the diet such as sodium acetate, as observed in small yellowfish (*Larimichthys polyactis*) [75]. The physiological significance of the higher ceramide contents in green *P. aibuhitensis* remains uncertain. It is speculated that this may be related to the special composition of the cuticle or other metabolic regulation demands. Further validation studies are needed.

### 4.6. Other Biochemical Compositions

Regarding other biochemical parameters, no significant differences in total cholesterol, triglycerides, and total protein content were observed between orange and green *P. aibuhitensis*. However, the green *P. aibuhitensis* had significantly higher total bile acid levels. Bile acids are one of the main components of bile, and they have functions such as emulsifying fats, promoting fat absorption [76,77], and enhancing immunity [78]. The elevated bile acid content in the green *P. aibuhitensis* may be associated with their higher demand for PUFA metabolism. Humans can endogenously synthesize bile acid and generally do not require exogenous supplementation in the diet, except in some disease statuses [79,80]. When utilized as supplements for aquafeed, careful evaluation of the dosage is essential. Studies by Su [76] and Jiang [81] have indicated that excessive bile acid supplementation may cause hepatopancreatic damage in shrimp and fish species.

In summary, the higher crude protein, lipid, and astaxanthin content and favorable n-3/n-6 PUFA ratio of the orange *P. aibuhitensis* suggests that this color type may be more suitable for dietary supplements and antioxidant functional products. The green *P. aibuhitensis*, with its higher contents of ceramides, n-6 PUFA, and bile acids, showed potential for targeted applications. This study was based solely on nutritional composition analysis, and the actual effects in humans or animal broodstocks require direct experimental validation.

## 5. Conclusions

Body color serves as an important indicator of nutritional quality in *P. aibuhitensis*. The orange *P. aibuhitensis* exhibit significant advantages in crude protein and lipid content, n-3/n-6 PUFA ratio, astaxanthin level, and triglyceride concentration, suggesting that they are more suitable for use as fish or shrimp broodstock diet as well as human nutritional supplements and antioxidant functional products. In contrast, the green *P. aibuhitensis* possess a distinct functional fatty acid profile, as well as higher levels of ceramides and bile acids, indicating their potential value for specific functional foods or applications targeting specific lipid requirements. These findings provide a critical theoretical basis for differentiating the application scenarios of the orange and green *P. aibuhitensis* based on body color. However, the above functional inferences require direct validation through feeding trials.

## Figures and Tables

**Table 1:** Proximate composition of the orange and green *P. aibuhitensis* (% wet weight).

Parameter	Moisture	Crude Protein	Crude Lipid	Crude Ash
Orange	79.04 ± 1.02	13.24 ± 0.61 **	2.55 ± 0.18 **	1.39 ± 0.07 **
Green	87.60 ± 0.71 **	8.16 ± 0.48	1.58 ± 0.11	0.93 ± 0.06

** means significant differences between the orange and green *P. aibuhitensis*, and the significance levels are *p* < 0.01.

## Data Availability

Data will be made available on request.

## Supplementary Materials

The following supporting information can be downloaded at https://www.mdpi.com/article/10.3390/biology15090706/s1. Figure S1. Lipid classification distribution of the orange and green *P. aibuhitensis*; Table S1: The number of molecular species and percentage for each class of lipids in the orange and green *P. aibuhitensis*.

## Abbreviations

The following abbreviations are used in this manuscript:

Abbreviation	Full Title
Fatty acids	
ARA	Arachidonic acid
DHA	Docosahexaenoic acid
EPA	Eicosapentaenoic acid
Amino acids	
Thr	Threonine
Val	Valine
Met	Methionine
Ile	Isoleucine
Leu	Leucine
Phe	Phenylalanine
Lys	Lysine
His	Histidine
Arg	Arginine
Tau	Taurine
Asp	Aspartic acid
Ser	Serine
Glu	Glutamic acid
Gly	Glycine
Ala	Alanine
Cys	Cysteine
Tyr	Tyrosine
Pro	Proline
EAAs	Essential amino acids
NEAAs	Non-essential amino acids
TAA	Total amino acids
Lipid molecules	
GP	Glycerophospholipid
PE	Phosphatidylethanolamine
PC	Phosphatidylcholine
CL	Cardiolipin
dMePE	Dimethylphosphatidylethanolamine
PS	Phosphatidylserine
MePC	Methylphosphocholine
LPC	Lysophosphatidylcholine
PEt	Phosphatidylethanol
MLCL	Monolyso cardiolipin
LPE	Lysophosphatidylethanolamine
LdMePE	Lysodimethylphosphatidylethanolamine
PI	Phosphatidylinositol
PG	Phosphatidylglycerol
BisMePA	Bis-methylphosphatidic acid
LPEt	Lysophosphatidylethanol
DLCL	Dilysocardiolipin
PA	Phosphatidic acid
BiotinylPE	Biotinylphosphatidylethanolamine
LPS	Lysophosphatidylserine
LPI	Lysophosphatidylinositol
GL	Glycerolipid
TG	Triglyceride
DG	Diglyceride
MGDG	Monogalactosyldiacylglycerol
DGMG	Digalactosyldiacylglycerol
MG	Monoglyceride
SP	Sphingolipid
Cer	Ceramide
Hex1Cer	Hexosylceramide
Hex2Cer	Dihexosylceramide
SPH	Sphingosine
SPHP	Sphingosine phosphate
ST	Sulfatide
GD1α	Ganglioside GD1α
LSM	Lyso sphingomyelin
phSM	Phosphosphingomyelin
CerP	Ceramide phosphate
GM3	Ganglioside GM3
Hex3Cer	Trihexosylceramide
FAs	Fatty acids
AcCa	Acyl carnitine
WE	Wax ester
OAHFA	(O-acyl)-1-hydroxy fatty acid
AEA	Anandamide
FA	Fatty acid
Co	Coenzyme
STs	Sterol lipids
CmE	Campesterol ester
ZyE	Zymosterol ester
ChE	Cholesterol ester
SiE	Sitosteryl ester
StE	Stearoyl ester
