# Supplementary material for: Comparison of the Chemical Composition of Different Body-Color Phenotypes of Perinereis aibuhitensis (Grube, 1878) (Annelida, Nereididae)
Source: Biology (Basel). 2026 Apr 30;15(9):706. doi: 10.3390/biology15090706 (PMC13162641; doi:10.3390/biology15090706)
Supplement: Supplementary file 1 [file biology-15-00706-s001.zip › biology-4251144-supplementary.pdf]

Supplementary Table S1. The number of molecular species and percentage for each class of lipids in the orange and green *P. aibuhitensis*

Lipid class	Number	Percentage/ %
**GP**		
PE	252	16.73
PC	199	13.21
CL	72	4.78
dMePE	35	2.32
PS	35	2.32
MePC	34	2.26
LPC	34	2.26
PEt	23	1.53
MLCL	14	0.93
LPE	14	0.93
LdMePE	10	0.66
PI	9	0.60
PG	8	0.53
BisMePA	4	0.27
LPEt	3	0.20
DLCL	3	0.20
PA	2	0.13
BiotinylPE	2	0.13
LPS	1	0.07
LPI	1	0.07
**GL**		
TG	255	16.93
DG	106	7.04
MGDG	11	0.73
DGMG	6	0.40

MG	6	0.40
**SP**		
Cer	188	12.48
Hex1Cer	67	4.45
Hex2Cer	5	0.33
SPH	4	0.27
SPHP	4	0.27
ST	3	0.20
GD1α	2	0.13
LSM	2	0.13
phSM	2	0.13
CerP	1	0.07
GM3	1	0.07
Hex3Cer	1	0.07
**FA**		
AcCa	16	1.06
WE	13	0.86
OAHFA	12	0.80
AEA	8	0.53
FA	4	0.27
Co	3	0.20
**ST**		
CmE	10	0.66
ZyE	10	0.66
ChE	5	0.33
SiE	5	0.33
StE	1	0.07

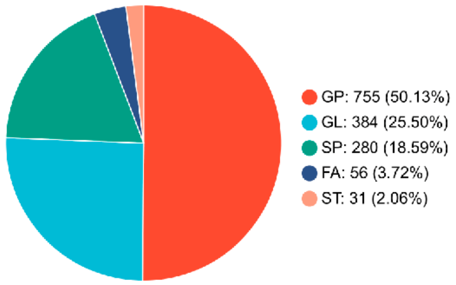

Supplementary Figure S1. Lipid classification distribution of the orange and green *P. aibuhitensis*
